# Stress Accumulation Induced by Ion Exchange for Synchronous Modulation of Mode and Wavelength in Microlasers

**DOI:** 10.1002/advs.75942

**Published:** 2026-05-30

**Authors:** Bingwang Yang, Lingling Sun, Jinhui Wang, Jitao Li, Peng Wan, Daning Shi, Caixia Kan, Mingming Jiang

**Affiliations:** ^1^ College of Physics MIIT Key Laboratory of Aerospace Information Sensing and Physics Key Laboratory for Intelligent Nano Materials and Devices Nanjing University of Aeronautics and Astronautics Nanjing P. R. China; ^2^ School of Physics and Telecommunications Engineering Zhoukou Normal University Zhoukou P. R. China

**Keywords:** CsPbCl_x_Br_3‐x_ microwire, ion exchange, single‐mode microlaser, strain accumulation, wavelength‐tunable

## Abstract

Achieving simultaneous and precise control over both the emission wavelength and lasing modes in laser architectures holds profound scientific significance for advancing integrated photonics. To date, no physical mechanism has been identified that enables the simultaneous modulation of lasing wavelength and output mode in perovskite microstructures through ion exchange. Here, we report a facile heating‐assisted vapor/solid anion exchange strategy to realize broadly tunable single‐mode microlasers based on pure‐phase single‐crystalline CsPbCl_x_Br_3‐x_ microwires. This approach seamlessly integrates bandgap engineering with intrinsic cavity design, where precise control over the halide composition enables continuous, linear wavelength tuning across a broad spectral range of 427–548 nm, the widest tuning range reported to date for Br/Cl anion‐exchange systems. Concurrently, lattice strain induced by ionic radius mismatch facilitates the formation of smooth slits within the microwires, which spatially partition the wirelike structure into mutually coupled Fabry‐Pérot (F‐P) subcavities, thereby enabling robust single‐mode polarized lasing via Vernier effect. Critically, this self‐contained approach maintains high crystalline quality, low threshold, and high quality (Q) factor, entirely circumventing the optical losses associated with external cavities or complex micro/nanofabrication. Combined with theoretical simulations that elucidate the mode selection and far‐field radiation mechanisms, this work establishes a transformative pathway toward multifunctional integrated photonics.

## Graphical abstract

This work demonstrates a paradigm‐shifting strategy to simultaneously achieve broadband wavelength tunability and single‐mode control within a single perovskite microwire structure. Using a facile heating‐assisted solid–solid anion‐exchange strategy, we synthesized phase‐pure CsPbCl_x_Br_3‐x_ microwires that exhibit the widest reported continuous lasing tunability (427–548 nm). Concurrently, ionic radii mismatch induces well‐controlled lattice distortion and in situ formation of atomically smooth slits. These intrinsic nanostructures partition the cavity into coupled Fabry–Pérot subcavities, enabling robust single‐mode polarized lasing via the Vernier‐like effect, which is achieved entirely without external cavities or lithographic processing. This self‐contained approach, validated by FDTD simulations, preserves pristine crystal quality, low thresholds, and high Q‐factor, establishing a transformative route to high‐coherence, broadly tunable single‐mode microlasers for advanced photonic integration.

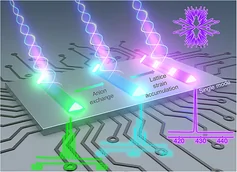

## 1 Introduction

Room‐temperature micro/nanolasers are recognized as fundamental components for high‐speed optical communications, high‐density data storage, and on‐chip spectral detection [1, 2, 3, 4]. Despite their considerable promise, the simultaneous realization of lasing behavior featuring precisely tunable emission wavelengths and single‐mode output is still regarded as a formidable challenge, which largely stems from the stringent requirements imposed on material properties and device architectures [5, 6, 7, 8]. Among the materials that have been investigated, halide perovskites are demonstrated to exhibit remarkable potential owing to their high absorption coefficients, excellent photoluminescence quantum yields, tunable bandgaps, and feasibility for microfabrication [9, 10, 11]. Bandgap modulation implemented in perovskite micro/nanostructures through anion exchange is regarded as a crucial approach to achieve the tunability of lasing output wavelength [12, 13, 14]. To date, most perovskite microstructures fabricated via ion exchange, especially microwires, are generally observed to exhibit gradient phase compositions instead of uniformly distributed single phases [15, 16]. Such gradient phase architectures are easily found to cause non‐uniform optical gain distribution and mode‐splitting, which are detrimental to the lasing performance. In addition, several drawbacks are still encountered, including a narrow tuning range and the lack of precise control over wavelength shifts to realize linear wavelength modulation.

To achieve perovskite single‐mode lasing, a variety of strategies have been developed by researchers. The most straightforward approach is to reduce the size of the laser cavity. However, this method has been confirmed to give rise to increased optical loss, which further induces a higher lasing threshold [17, 18]. Other proposed schemes include the incorporation of low‐dimensional perovskite micro/nanostructures into distributed Bragg reflector or distributed feedback structures, as well as the construction of coupled cavities on the basis of the Vernier effect or parity‐time symmetry [19, 20, 21, 22]. Among these strategies, the implementation of groove structures to achieve controllable modes in micro/nanolasers has been reported [23, 24]. These experimental strategies, by which the lasing modes are modulated via structural design, undoubtedly represent significant breakthroughs. Nevertheless, the introduction of an external cavity inevitably results in the degradation of the microcavity Q factor. Such top‐down approaches typically require sophisticated operations, including photolithography, etching, focused ion beam, and femtosecond laser processing [25, 26, 27, 28]. These techniques are prone to introducing defects or causing contamination during fabrication, thereby degrading the crystalline quality and optical performance of the material. Notably, the above methods are limited to controlling the lasing mode and fail to achieve tunable emission wavelength. Therefore, the utilization of a facile, non‐destructive strategy to simultaneously realize lasing wavelength tuning and single‐mode output is considered a critical technical challenge for the fabrication of high‐performance micro/nanolasers.

In this work, a facile heating‐assisted vapor/solid halide ion exchange method was employed to prepare CsPbCl_x_Br_3‐x_ (CPCB) single‐crystal microwires with natural slit structures, by which simultaneous modulation of the lasing wavelength and output mode was achieved. The obtained microwires exhibit a uniform composition and a pure‐phase structure, ensuring the stability of the lasing output. By controlling the ion‐exchange time, the lasing emission wavelength can be continuously tuned over a broad spectral range from 427 to 548 nm. With extended ion‐exchange duration, intrinsic slit‐like structures derived from stress accumulation emerged spontaneously. Coupled microcavities were constructed between such structures and microwires, enabling the eventual achievement of single‐mode lasing. The underlying physical mechanism was elucidated via theoretical modeling, which reveals that high‐performance single‐mode F‐P lasing is realized through a slit‐induced multiple‐cavity coupling effect within the microwires. This effect enables effective single‐mode selection for a large‐size microcavity, eliminating the requirement for an external cavity. This work innovatively transforms the stress accumulation generated during the ion exchange process into an effective means for modulating lasing modes, offering a viable approach for the design of multifunctional integrated optoelectronic devices.

## 2 Results and Discussions

**FIGURE 1:**
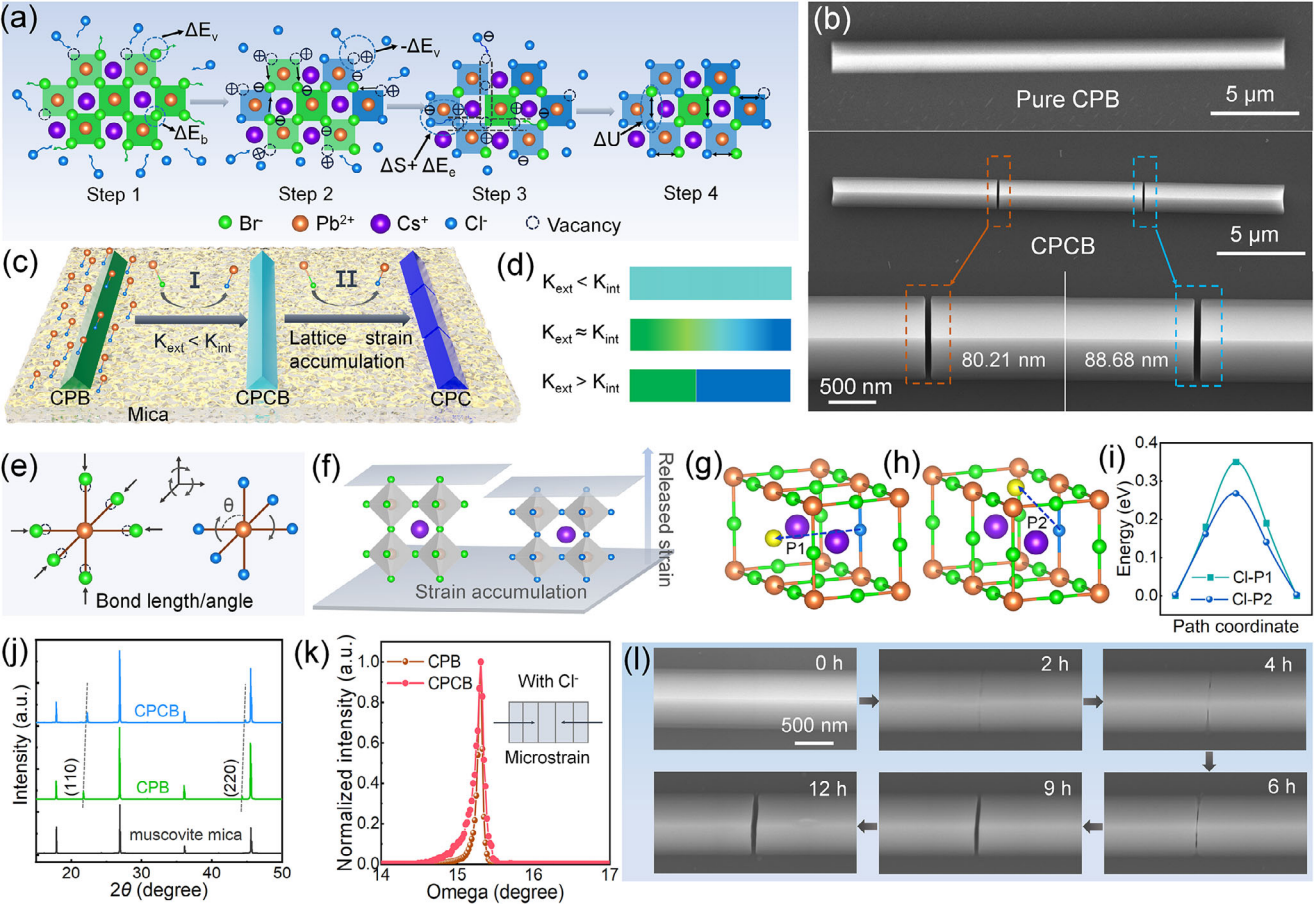
Fabrication of CPCB microwires and analysis of the formation mechanism of internal slit structures. (a) Schematic illustration of the in situ exchange channels promoting uniform anion exchange. (b) SEM images of the microwires before and after anion exchange at a heating temperature of 110°C. (c) Schematic diagram of the slit formation process within the microwires. (d) Schematic diagrams of anion exchange results under different conditions. (e) At the atomic scale, halogen ion substitution leads to variations in Pb─X bond lengths and X─Pb─X bond angles. (f) Schematic illustration of strain relaxation realized via the introduction of small‐sized ions. (g) Schematic diagram showing the migration pathways of Cl^−^ ions along the (100) direction. (h) Schematic diagram showing the migration pathways of Cl^−^ ions along the (110) direction. (i) Energy distributions at different positions during Cl^−^ ion migration along paths P1 and P2, respectively. (j) XRD patterns of the samples before and after anion exchange. (k) Rocking curves of the (110) diffraction peak for the samples before and after anion exchange; the inset presents a schematic illustration of the microstrain induced by Cl^−^ incorporation. (l) The slit width gradually increases with prolonged reaction time.

The anion‐exchange method has been widely employed as a core approach for modulating lasing emission wavelengths in perovskite lasers. Its microscopic process generally involves surface anion desorption and vacancy generation, infiltration of foreign anions, and subsequent uniform ion distribution and replacement [29]. To gain an in‐depth understanding of the microscopic dynamics of anion exchange, a kinetic model is established to clarify the relationship between the energy evolution and anion migration throughout the anion‐exchange process. As illustrated in Figure 1a, the pristine perovskite lattice initially resides in a relatively stable state. During anion exchange, the desorption of surface Br^−^ must overcome an energy barrier (ΔE_b_) associated with its bonding configuration (Step 1). This energy barrier for Br^−^ desorption (ΔE_v_) is subsequently reduced through the introduction of Cl^−^, which substitutes for the surface Br^−^. As depicted in Step 2, after a large number of anion vacancies are generated, Cl^−^ ions driven by binding energy are more readily incorporated onto the perovskite surface [30]. As the reaction proceeds continuously, surface Br^−^ ions are gradually desorbed, and the vacancy concentration is increased, leading to the formation of a compositional gradient between the interior and the surface of the sample. Such an unbalanced distribution of vacancies and anions induces a local internal electric field inside the perovskite, thereby raising the total energy of the system. To preserve a low‐energy state, Br^−^ ions are driven to diffuse spontaneously toward the surface under the action of the internal electric field (ΔE_e_) and system entropy (Δ_s_), while vacancies are inclined to be uniformly distributed. In this way, the system energy is balanced, and the internal electric field is weakened correspondingly [31]. These spontaneous processes slightly increase the system entropy and reduce the total energy, by which the system is maintained in a relatively stable state. Meanwhile, the diffusion of vacancies toward the interior provides a rapid pathway for the further exchange of Cl^−^ ions (Step 3). Ultimately, driven by the internal energy of the system (ΔU), a uniform distribution of Cl^−^ is achieved (Step 4), and single‐crystal CPCB microwires are obtained.

Based on the above model, we fabricated CPCB microwires with uniform composition and pure‐phase structure by precisely controlling the reaction temperature and time during the anion exchange process. The CsPbBr_3_ (CPB) microwires were first synthesized via chemical vapor deposition (CVD), with an optical photograph of the as‐prepared CPB microwires shown in Figure S1. The CPB microwires were placed together with PbCl_2_ powder into a sealed Petri dish, which was subsequently heated on a hot stage to conduct the anion‐exchange reaction. Single‐crystal CPCB microwires with tunable halogen composition ratios could be obtained by adjusting the reaction conditions, and the simple fabrication setup is displayed in Figure S2. Notably, with prolonged exchange time, fractured slits were observed within the pre‐prepared CPCB microwires. SEM images of the microwire before and after the anion exchange process are presented in Figure 1b, where a well‐defined slit morphology with smooth and straight edges is clearly observed. The slit width was measured to be approximately 80–90 nm, by which the whole microwire was divided into multiple F‑P microcavities. According to relevant literature, such ultra‐narrow air gaps are known to induce a coupling effect between microcavities, thereby enabling single‐mode lasing output [21, 32]. Figure S3a presents the optical images of the microwires before and after anion exchange, and the formation of slits inside the microwires can be clearly observed after Br^−^ is substituted by Cl^−^. The SEM images and EDS elemental mappings of the anion‐exchanged samples are shown in Figures S3b and S4, which further confirm the presence of fractured slits within the microwires. Interestingly, slits with different numbers were observed to emerge on the microwires as the ion‐exchange duration increased, as depicted in Figure S5. These microwire samples with different slit features exert a tunable effect on the lasing output performance.

Next, the formation process of the slits was investigated. As illustrated in Figure 1c, thermal activation drives the interdiffusion of halide species at the microwire interface, where Cl^−^ ions from PbCl_2_ interdiffuse with Br^−^ ions from the CPB matrix. This process enables compositional intermixing, which is governed by concentration gradients at elevated temperatures [14]. Given that the layered structure of PbCl_2_ inherently suppresses the outward diffusion of Cl^−^ ions, the rate of interfacial halide exchange remains relatively low. In contrast, Cl^−^ ions that have penetrated into the bulk crystals undergo rapid, homogeneous redistribution at elevated temperatures, as enabled by thermally activated diffusion pathways. As shown in the above analysis, a pure‐phase halide product with homogeneous elemental distribution can be reproducibly obtained under conditions where the internal halide exchange rate dominates the interfacial mass transport [15, 33]. This kinetically favorable regime provides the thermodynamic driving force required to suppress phase segregation, yielding a uniform and structurally pure microstructure. Because Cl^−^ has a smaller ionic radius than Br^−^, the ion exchange process induces lattice contraction and distortion, which in turn generates localized microstresses within the crystal lattice [34, 35]. As the exchange reaction proceeds, the progressive accumulation of these microstresses ultimately results in the spontaneous fracture of the microwires. As schematically illustrated in Figure 1d, the interplay between internal exchange (K_(int)_) and external interdiffusion (K_(ext)_) rates governs the halide redistribution behavior within the microwire. A balanced rate ratio (K_(int)_ ≈ K_(ext)_) promotes the formation of compositionally graded structures. In contrast, when K_(int)_ < K_(ext)_, this kinetic regime preferentially yields well‐defined sharp heterojunctions. Conversely, when K_(int)_ dominates over K_(ext)_, rapid redistribution of Cl^−^ ions is enabled, resulting in complete compositional homogenization and thus highly stable lasing performance.

The formation mechanism of the slits was further explored. Given that the ionic radius of Cl^−^ is smaller than that of Br^−^, the Pb─X bond length is shortened upon Cl^−^ substitution (Figure 1e), consequently inducing local lattice strain [35, 36]. As the exchange time is prolonged, the strain progressively accumulates and is ultimately released through the generation of microcracks, resulting in slit‐like fractures. To relieve the geometric frustration imposed by the incorporation of different halide ions, the bond lengths and angles within the [PbX_6_] octahedral framework undergo systematic contraction or elongation. This lattice distortion induces crystallographic mismatch, which serves as the direct source of local compressive stress. The accumulation of this stress is subsequently relieved through the formation of these slits, as evidenced in Figure 1f. Additionally, the vacancy‐assisted diffusion mechanism was investigated at the atomic scale. As illustrated in Figure 1g,h, P1 and P2 correspond to the migration pathways of Cl vacancies along the (100) and (110) directions, respectively. The energy profiles for Cl ion migration along the two pathways are displayed in Figure 1i. The findings demonstrate that the migration activation energy along the (110) direction is lower, indicating that the (110) direction acts as the dominant pathway for Cl^−^ vacancy migration. Theoretical calculations indicate that the relatively low migration barrier of Cl ions facilitates their migration and diffusion, which is conducive to the formation of compositionally uniform perovskite microwires [13, 33, 37]. XPS characterization in Figure S6 reveals that, with prolonged anion exchange time, Br^−^ in CPB was gradually substituted by Cl^−^. As evidenced by the XRD analysis (Figure 1j), the incorporation of smaller Cl^−^ ions leads to a distinct shift of the (110) and (220) diffraction peaks toward higher angles [13, 38]. This shift is attributed to a reduction in the corresponding interplanar spacing, directly indicating the presence of residual compressive stress within the material [14, 39]. The compressive strain induced by small‐sized Cl^−^ ions gives rise to lattice contraction, resulting in an inhomogeneous stress distribution. The rocking curve in Figure 1k shows that the full width at half maximum (FWHM) of the (110) diffraction peak for the anion‐exchanged sample is significantly broadened, confirming that the incorporation of Cl^−^ introduces microstrain into the CPB crystal structure [39, 40]. Furthermore, the considerable thermal expansion coefficient mismatch between perovskite microwires and mica substrates should also be taken into account, which may likewise give rise to stress accumulation [34, 41]. To this end, additional experiments were conducted to rule out the possibility that slit formation originates from the thermal expansion coefficient difference between adjacent layers (Figure S7). Figure 1l displays the slit formation on perovskite microwires from the same batch at a heating temperature of 180°C with varied ion exchange durations. It is observed that the slit width exhibits a monotonically increasing trend with prolonged exchange time. Such controllable structural evolution may offer a feasible approach for modulating the resonant modes within the microcavity.

**FIGURE 2:**
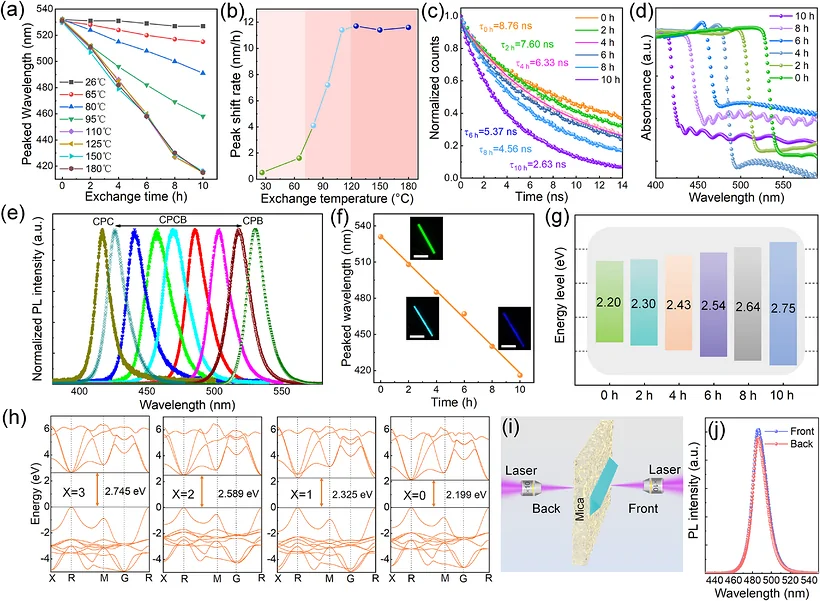
Optical characterizations of CPCB microwires. (a) Dependence of lasing emission peak position on ion‑exchange duration at different reaction temperatures. (b) Shift rate of the lasing emission peaks. (c) TRPL and (d) absorption spectra of the samples at different ion‑exchange durations. (e) Evolution of PL spectra of the as‐fabricated microwires. (f) Linear relationship between PL peak position and reaction time, scale bar: 5 µm. (g) Variation of the sample bandgap value with prolonged anion exchange time. (h) Simulated electronic band structure. (i) Schematic illustration of the testing method for compositional uniformity of the microwires. (j) PL spectra of the top and bottom regions of the microwires.

The ion‐exchange rate depends critically on the reaction temperature, which underscores that temperature acts as the dominant kinetic parameter governing the entire halide exchange process [42]. Figure 2a shows the evolution of the PL peak positions of CPCB microwires as a function of exchange time at temperatures ranging from 26 to 180°C. The results confirm that, while the peak positions exhibit a continuous blueshift across all investigated temperatures, the rate of the exchange process exhibits a pronounced dependence on the thermal conditions. In the low‐temperature regime (<65°C), the exchange proceeds at a markedly sluggish rate, with a blueshift of only ∼16 nm observed after 10 h. In contrast, elevating the temperature to the 80°C–110°C range results in a significant acceleration of the exchange rate. At 110°C, the PL peak position was blueshifted from 530 to 415 nm within 10 h, and the maximum spectral tuning range was achieved, indicating that the most pronounced ion exchange kinetics occurred in this temperature regime. As the temperature was further elevated, the rate of blueshift ceased to increase, suggesting that the process was likely limited by diffusion. As shown in Figure 2b, the halide exchange rate unequivocally peaks in the 80°C–110°C temperature window. This confirms that the reaction temperature is the dominant factor governing the extent of the halide exchange reaction. The homogeneity of the compositional distribution within the microwires is critically determined by precise control of the interfacial halide exchange rate. An excessively high reaction temperature drives this rate into an uncontrollable regime, where severe compositional inhomogeneities are readily induced. Accordingly, to maintain well‐defined, kinetically controlled reaction conditions, all subsequent halide exchange experiments were conducted at a fixed temperature of 110°C.

The influence of compositional variation on the optical properties of the samples was further investigated. As shown by the time‐resolved photoluminescence (TRPL) curves in Figure 2c, a shortening trend in the fluorescence lifetime was observed with increasing Cl^−^ content. This phenomenon was attributed to the lattice mismatch and halogen vacancy defects introduced during the anion exchange process [43]. As demonstrated by the absorption spectra in Figure 2d, the absorption edge was gradually shifted toward the short‐wavelength region with the prolongation of the exchange time. Figure 2e shows that the PL spectra exhibited a distinct blueshift with increasing Cl^−^ content, which was ascribed to the gradual widening of the alloy band gap [37, 44]. As further revealed in Figure 2f, the PL peak wavelength exhibited a favorable linear shift trend with reaction time at a heating temperature of 110°C, indicating that wavelength modulation could be realized by precisely controlling the reaction time. The influence of anion exchange time on the bandgap value of the samples is presented in Figure 2g. After Br atoms were substituted by Cl atoms, the conduction band minimum was shifted upward, and the valence band maximum was shifted downward, leading to an enlarged bandgap value. This trend establishes a direct agreement with the theoretical predictions (Figure 2h), thereby substantiating the validity of our proposed halide exchange strategy. To confirm the compositional uniformity of the CPCB microwires, micro‐PL spectra were collected from both the top and bottom of the microwire, as presented in Figure 2i. The results in Figure 2j revealed highly consistent PL peak positions between the two sides, indicating that Cl^−^ and Br^−^ ions can diffuse rapidly and uniformly within the crystal lattice [45], thereby leading to the formation of a single‐phase structure rather than a core–shell or graded composition distribution. Overall, the reaction rate of the ion‐exchange process was dominated by the controlled evaporation of PbCl_2_. Thus, pure‐phase microwires with varied compositional ratios yet uniform elemental distribution could be obtained by precisely regulating the reaction time. The resultant compositionally homogeneous, pure‐phase structures are conducive to reducing optical loss and suppressing carrier localization, thereby laying a critical material foundation for achieving stable stimulated emission.

**FIGURE 3:**
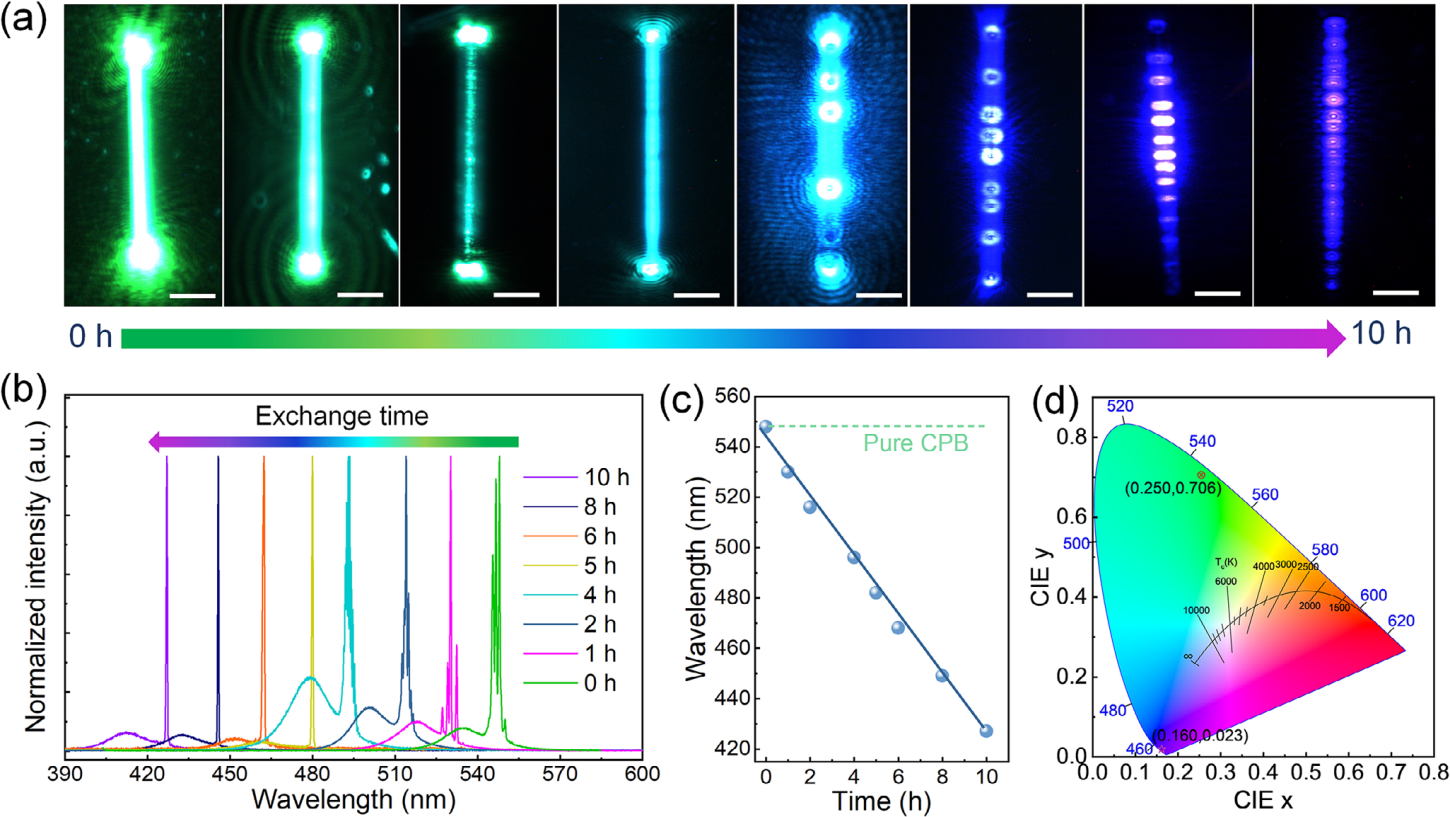
Synchronously tunable lasing wavelength and modes of CPCB microwires. (a) Dark‐field luminescence images of microwires obtained at different reaction times, scale bar: 5 µm. (b) Lasing spectra of microwires at different exchange durations. (c) Evolution of the lasing peak positions of microwires with exchange duration. (d) Chromaticity diagram illustrating the continuous variation of lasing emission color with reaction time.

Leveraging the bandgap tunability of the material system, we demonstrate tunable lasing emission from CPCB microwires through systematic investigation of their optically pumped lasing characteristics. The transition to the lasing regime is evidenced by the dark‐field luminescence image captured above the lasing threshold, as depicted in Figure 3a. As the halide exchange reaction proceeds, the emission spectra exhibit a continuous blueshift, with the lasing wavelength gradually tuning from the green to the blue–violet spectral region. Notably, strong mode confinement and coherent emission at the microwire termini are observed throughout the entire tuning range. This is a characteristic signature of longitudinal mode oscillation in F‐P resonators [46]. As the halide exchange proceeds, slit architectures are formed along the microwires, effectively segmenting them into multiple F‐P microcavities. Furthermore, unambiguous lasing signals are detected in the slit regions. This phenomenon stems from the evanescent coupling between neighboring microcavities, which enables coherent emission in the otherwise uncoupled interstitial regions. The lasing spectra depicted in Figure 3b demonstrated that the lasing emission wavelength underwent a blueshift with prolonged reaction time. Interestingly, single‐mode lasing emission was observed at approximately 481, 464, 449, and 427 nm, respectively, starting from 5 h of anion exchange. This behavior was presumably attributed to the coupling between multiple F‐P microcavities, essentially arising from the Vernier effect. As shown in Figure 3c, a positive correlation was observed between the lasing peak position and the anion exchange time, with the emission wavelength tunable across a range of 548–427 nm. This tuning range is among the most extensive reported for Br/Cl exchange systems [13, 14, 47]. The CIE chromaticity coordinates (0.250, 0.706) and (0.160, 0.023) in Figure 3d intuitively reflected the evolution trend of the emission color with anion‐exchange duration. These results demonstrate that by adjusting the reaction time, the lasing color of CPCB microwires can be flexibly tuned over a broad spectral range. Importantly, effective modulation of the lasing mode was realized while achieving continuous tuning of the lasing peak position, which offered a research strategy for obtaining stable single‐mode lasing output in the target waveband. These results demonstrated that the as‐fabricated perovskite microstructures possessed promising applications in color/white light‐emitting diodes and mode‐tunable lasers. The reliability of the halide exchange protocol was confirmed by benchmarking it against directly synthesized reference materials. Pristine CVD‐grown CsPbCl_3_ microwires generated lasing emission at 426 nm (Figure S8). In comparison, CPCB microwires acquired from a 10‐h halide exchange process showed lasing at 427 nm. The lasing output wavelengths of the two samples were found to be almost identical, which verified the high sufficiency and reliability of the proposed anion‐exchange approach.

**FIGURE 4:**
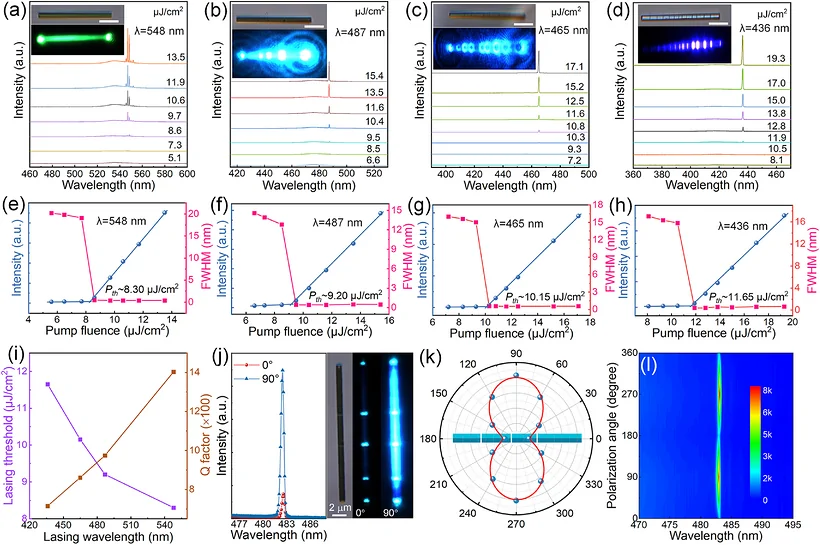
Lasing performance characterizations of CPCB microwires at different emission wavelengths. Power‐dependent PL spectra of the typical lasing emission were recorded at (a) 548 nm, (b) 487 nm, (c) 465 nm, and (d) 436 nm, scale bar: 5 µm. (e–h) Evolution of PL intensity and FWHM as functions of pump energy density at various lasing emission wavelengths. (i) Plots of lasing threshold and Q‐factor as functions of lasing emission wavelengths. (j) Polarized lasing spectra, optical photographs, and dark‐field luminescence images of the sample at angles of 90° and 0°. (k) Polarized lasing output performance of the sample within the polarization angle range of 0–360°. (l) Modulation characteristics of the lasing intensity of the sample under excitation at different polarization angles.

Utilizing compositionally uniform, pure‐phase perovskite microwires as self‐formed gain media and resonant cavities, we realize broadband and continuously tunable lasing emission together with stable robust single‐mode operation. The crucial factor enabling this performance is the generation of nanoscale slits during halide exchange. We identify these slit structures as critical for the simultaneous and independent manipulation of lasing wavelength and mode selection. The evolution of lasing spectra at emission wavelengths of 548, 487, 465, and 436 nm with increasing pump fluence is presented in Figure 4a–d, respectively. An evident spectral transition from wideband spontaneous radiation to stimulated emission was identified across all spectra with rising pump fluence, alongside the appearance of numerous narrow resonant peaks. Notably, coupled microcavities stably sustained single‐mode lasing across diverse elevated pump fluences, with corresponding lasing wavelengths fixed at 487, 465, and 436 nm. Notably, for the lasing mode at ∼ 465 nm, a side‐mode suppression ratio of up to 20.0 dB was achieved at a pump fluence of 17.1 µJ/cm^2^, surpassing those reported for conventional F‐P single‐mode lasers in prior literature [48, 49]. These results demonstrate that the incorporation of the slits formed within the microwires not only enables tunable lasing wavelengths but also facilitates effective mode control, enabling high‐quality single‐mode lasing. As shown in Figure 4e–h, the lasing thresholds for the emission wavelengths at 548, 487, 465, and 436 nm were determined to be 8.30, 9.20, 10.15, and 11.65 µJ/cm^2^, respectively. A gradual increase in threshold was observed with the blueshift of the emission wavelength, indicating that the incorporation of Cl^−^ ions introduces nonradiative recombination centers, which reduce the photoluminescence quantum efficiency and consequently weaken the optical gain. Meanwhile, a gradual decrease in the Q‐factor of the CPCB microwires was observed as the emission wavelength blue‐shifted (Figure S9), which further suggested that vacancy defects deteriorate the optical resonant confinement capability of the microcavities. The evolution of the lasing threshold and Q‐factor with the blueshift of the emission wavelength is directly illustrated in Figure 4i. Notably, it was found experimentally that some slit‐containing microwires failed to achieve single‐mode output. As depicted in Figure S10a, the CPCB microwire featuring a slit with a width of approximately 135 nm was observed, whereas single‐mode lasing output was not achieved (Figure S10b). This phenomenon could be attributed to the excessively large width of the air gap, which suppressed optical transmission between adjacent microcavities and gave rise to weakened coupling strength [21]. Insufficient coupling strength resulted in decreased mode suppression efficiency, ultimately giving rise to multimode output. Conversely, single‐mode lasing was successfully achieved in the microwire featuring two narrower slits with widths of 81 and 96 nm Figure S10c,d. Therefore, the key to realizing single‐mode lasing in slit‐containing microwires lies in maintaining a sufficiently narrow air gap to ensure adequate coupling efficiency.

To systematically investigate the polarization‐resolved characteristics of the slit‐containing CPCB microlasers, we performed optical pumping experiments by varying the excitation polarization angle. The emission spectra acquired at different polarization angles *θ* are displayed in Figure 4j. The emission intensity at *θ* = 90° was approximately six times higher than that at *θ* = 0°. This underscores the dominance of the transverse electric mode in the lasing emission [50]. This pronounced angular dependence was further corroborated by dark‐field luminescence imaging. The imaging revealed substantially stronger emission along the *θ* = 90° direction. Collectively, these observations provide definitive evidence for the strongly anisotropic emission characteristics inherent to the microlaser. The polar plot of lasing intensity vs. polarization angle was depicted in detail in Figure 4k, which directly demonstrated that the maximum lasing intensity occurred near 90° and 270°. The degree of polarization (DOP) was calculated to be approximately 0.71 using the equation DOP = (*I_max_ – I_min_*)/(*I_max_ + I_min_*), indicating that the lasing emission exhibited a highly polarized state. Periodic modulation of the output intensity with the excitation polarization angle at the lasing peak of approximately 482 nm was presented in Figure 4l. A stable and repeatable periodic fluctuation of the lasing output intensity was observed with the continuous variation of the polarization angle, which further validated the excellent polarization operational stability of the CPCB microwire microlasers with slit structures. These findings demonstrate that the slit‐containing CPCB microwires are capable of simultaneously achieving lasing intensity modulation and high‐purity polarized emission, holding promise for applications in emerging multifunctional optoelectronic devices.

**FIGURE 5:**
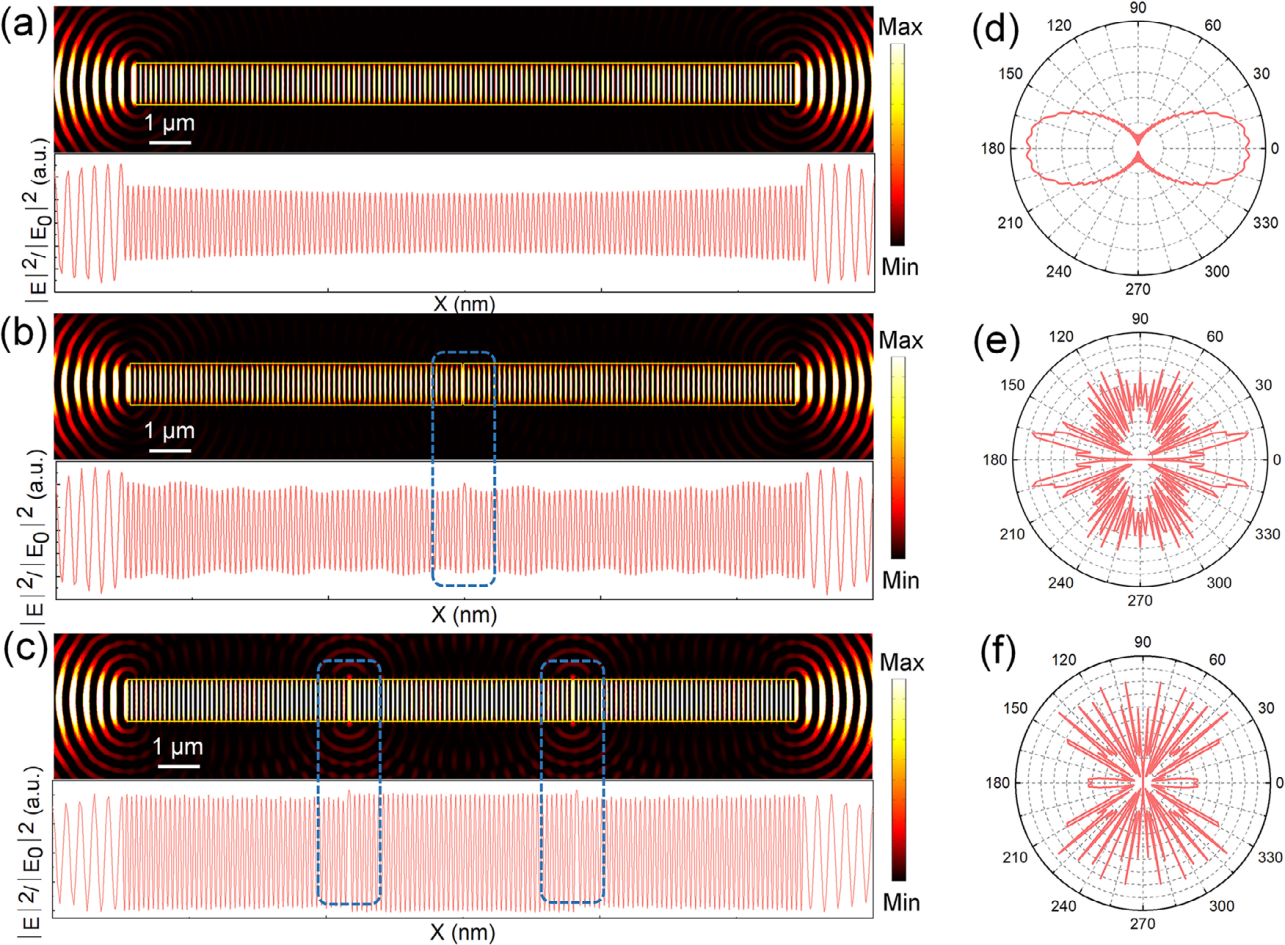
FDTD simulation of CPCB microwires with slit structures. The simulated electric field distributions and the corresponding spectral data of the field distribution were obtained for microwires (a) without slits, (b) with one slit, and (c) with two slits. The far‐field radiation patterns are presented for microwires (d) without slits, (e) with one slit, and (f) with two slits.

To further investigate the directionality of lasing emission in CPCB microwires with slit structures, the finite‐difference time‐domain (FDTD) method was adopted to systematically simulate and compare the radiation characteristics of microwires with different slit numbers. For the slit‐free microwire microcavity, it was clearly observed that the optical field was well confined within the cavity. The resonant modes were reflected back and forth along the longitudinal direction and eventually leaked out from the end facets, as illustrated in Figure 5a. Figure S11 showed the enlarged electric field distribution at the end faces of the microwire and the magnified spectral profile. When a slit was introduced into the microcavity, the entire cavity could be regarded as multiple F‐P sub‐cavities arranged along the axial direction. Photons were not only emitted from the end facets but also leaked out through the slit regions. The simulation results further revealed that the microcavity exhibited a large free spectral range in the slit region, with the corresponding simulated electric field distribution and spectral data presented in Figure 5b,c. The enlarged electric field distributions at both the end faces and the slits of the microwires, together with the magnified spectral profiles at the slits, are presented in Figure S12. Benefiting from the above characteristics, single‐mode lasing was successfully realized in microwires embedded with slit structures. It is concluded that the single‐mode selection achieved via the introduction of slit structures essentially originates from the coupling effect between multiple cleaved F‐P sub‐cavities. The far‐field radiation simulations in Figure 5d–f showed that the microwire without ion exchange exhibited distinct directional lasing along 0° and 180°, with light emitted from both ends of the microwire. After a single slit was introduced, coupling arose between the two independent F‐P sub‐cavities, leading to remarkably enhanced emission at the slit. The radiation was concentrated near 90° and 270° with high directivity. For the microwire with two slits, the three independent F‐P sub‐cavities interacted at the two slit regions, where lasing emission was generated. The two lasing sources interfered with each other, leading to intense radiation along 45°, 135°, 225°, and 315° in the far‐field distribution, whereas the original radiation at 90° and 270° was weakened. Therefore, superior emission directivity can be achieved in slit‐free and single‐slit structures, whereas the influence of interference must be considered for microwires with two slits or more. The multi‐directional radiation demonstrated by such slit‐based single‐mode microlasers may show promising application potential in scenarios including multi‐angle directional emission, multi‐view display, and rapid parallel detection. Furthermore, stability is one of the key factors for perovskite micro‐nano laser devices toward practical applications. Through a series of stability tests, the as‐prepared samples were confirmed to exhibit excellent resistance to irradiation damage and humidity stability (Figure S13).

## 3 Conclusion

In summary, this work presents a paradigm shift in the simultaneous control of lasing wavelength and mode within single‐crystal perovskite microstructures during the ion exchange process, resolving a long‐standing challenge in integrated photonics. By developing a facile heating‐assisted vapor/solid halide exchange strategy, we synthesize pure‐phase, compositionally uniform CPCB single‐crystalline microwires with in situ formed slit structures. This approach seamlessly integrates bandgap engineering with intrinsic cavity design, enabling continuously tunable lasing across an unprecedented spectral range of 427–548 nm, the broadest reported for Br/Cl exchange systems to date. Critically, we reveal that stress accumulation from ionic radius mismatch induces atomic‐scale lattice distortion and the spontaneous formation of atomically smooth slits. These intrinsic slits partition the microwire into coupled F‐P sub‐cavities, enabling external‐cavity‐free, highly polarized single‐mode lasing via the Vernier effect. Unlike conventional top‐down fabrication, this nondestructive strategy preserves exceptional crystalline integrity and high Q‐factor, achieving low‐threshold and robust single‐mode output. Supported by FDTD simulations elucidating the intercavity coupling mechanism and far‐field directionality, this research effectively addresses the long‐standing challenge of reconciling lasing wavelength tunability with mode control in micro/nanolasers, thereby establishing a scalable paradigm for high‐performance multifunctional coherent light sources that catalyzes transformative advances in chip‐integrated photonics.

## 4 Experimental Section

### 4.1 Materials

PbBr_2_, CsBr, and PbCl_2_ were purchased from Shanghai Macklin Biochemical Technology Co., Ltd. All reagents were used as received without further purification.

### 4.2 Fabrication of CPCB Microwires and Slit Structures

The CPB microwires were placed together with an appropriate amount of PbCl_2_ powder in a sealed Petri dish and subjected to heat treatment on a hot plate, enabling the CPB microwires to undergo halide ion exchange with the evaporated PbCl_2_. The distance between PbCl_2_ and CPB microwires is approximately 1 cm. By controlling the heating temperature and the duration of the halide ion exchange, CPCB microwires with varying compositional ratios were obtained. With the increase of ion‐exchange time, slit structures were formed on the CPCB microwires due to the stress accumulation induced by ion exchange.

### 4.3 Structural and Morphology Characterization

The morphological features of the fabricated CPB and CPBC microwires with slits were examined using the SEM and optical microscopy. XRD patterns were acquired employing a Rigaku D/max 2550 X‐ray diffractometer with Cu Kα radiation. The elemental compositions of the samples were determined by EDS. To identify the valence states in the CPBC microwires, XPS analysis was conducted using a Thermo Scientific Escalab‐250 instrument equipped with an Al Kα source operating at 1486.6 eV.

### 4.4 Optical Performance Characterization

The optical performance of the fabricated samples was examined using absorption spectroscopy (UV‐3150) and PL spectroscopy. Fluorescence images of the samples were acquired using a fluorescence imaging microscope (E3‐iFL, Nanjing Metatest Optoelectronics Co., Ltd.). TRPL spectra were acquired with the streak camera. A pumped laser (wavelength: 325 nm; pulse duration: 100 fs; repetition rate: 1 kHz) was generated by an Optical Parametric Amplifier combined with the coherent Ti: sapphire laser. The laser beam was then focused onto the sample using the micro‐PL (Olympus). The polarization properties of the samples were determined by the microscope equipped with an anisotropy module. The excitation process was performed top‐down using a femtosecond pulsed laser (wavelength 325 nm), and the emitted light was collected by the objective lens after passing through a polarizer.

### 4.5 Numerical Simulation

In the simulation, the length of the microwires was set to 16 µm, with a thickness of 850 nm. Under transverse electric mode, a search for eigenmodes of the cavity was performed over a wavelength range spanning from 476 to 540 nm, which was consistent with the experimentally measured lasing wavelengths. The refractive index of the perovskite was taken as 2.51, while that of the mica substrate was set to 1.59 [51]. During the simulation, three‐component vector calculations of the electromagnetic field in the frequency domain were performed, and far‐field monitors were placed in the space surrounding the microwire to capture the radiation distribution. The mesh sizes were set to λ/15 in the structure region and λ/6 in the air region, where λ denotes the wavelength. Perfectly matched layers were employed at the outer boundaries, applying mapped boundary conditions to minimize reflection errors. According to the target wavelengths, calculations were conducted around the eigenfrequencies identified during the mode search, while the symmetry of the structure was exploited to reduce computational complexity.

## Author Contributions

**Jitao Li**: methodology, validation, formal analysis. **Lingling Sun**: methodology, validation, visualization. **Peng Wan**: investigation, visualization. **Daning Shi**: supervision, funding acquisition. **Jinhui Wang**: data curation, formal analysis. **Bingwang Yang**: conceptualization, investigation, writing – original draft, project administration, validation. **Caixia Kan**: supervision, formal analysis. **Mingming Jiang**: conceptualization, investigation, funding acquisition, writing – review and editing, project administration, supervision.

## Funding

This study is supported by the National Natural Science Foundation of China (Grant Nos. 12374257 and 62404101), and the Postgraduate Research & Practice Innovation Program of Jiangsu Province (KYCX25_0638).

## Conflicts of Interest

The authors declare no conflicts of interest.

## Supporting information




**Supporting File**: advs75942‐sup‐0001‐SuppMat.docx.

## Data Availability

The data that support the findings of this study are available from the corresponding author upon reasonable request.
