# Supplementary material for: Stress Accumulation Induced by Ion Exchange for Synchronous Modulation of Mode and Wavelength in Microlasers
Source: Adv Sci (Weinh). 2026 May 30:e75942. Online ahead of print. doi: 10.1002/advs.75942 (PMC13336736; doi:10.1002/advs.75942)
Supplement: Supplementary file 1 — Supporting File: advs75942‐sup‐0001‐SuppMat.docx. [file ADVS-9999-e75942-s001.docx]

Supporting Information

Stress Accumulation Induced by Ion Exchange for Synchronous Modulation of Mode and Wavelength in Microlasers

*Bingwang Yang, Lingling Sun, Jinhui Wang, Jitao Li, Peng Wan, Daning Shi, Caixia Kan, and Mingming Jiang**

B. Yang, J. Wang, Prof. P. Wan, Prof. D. Shi, Prof. C. Kan, and Prof. M. Jiang

College of Physics

MIIT Key Laboratory of Aerospace Information Sensing and Physics

Key Laboratory for Intelligent Nano Materials and Devices

Nanjing University of Aeronautics and Astronautics

Nanjing 211106, P. R. China.

E-mail: mmjiang@nuaa.edu.cn (M. Jiang)

L. Sun, J. Li

School of Physics and Telecommunications Engineering

Zhoukou Normal University

Zhoukou 466001, P. R. China.


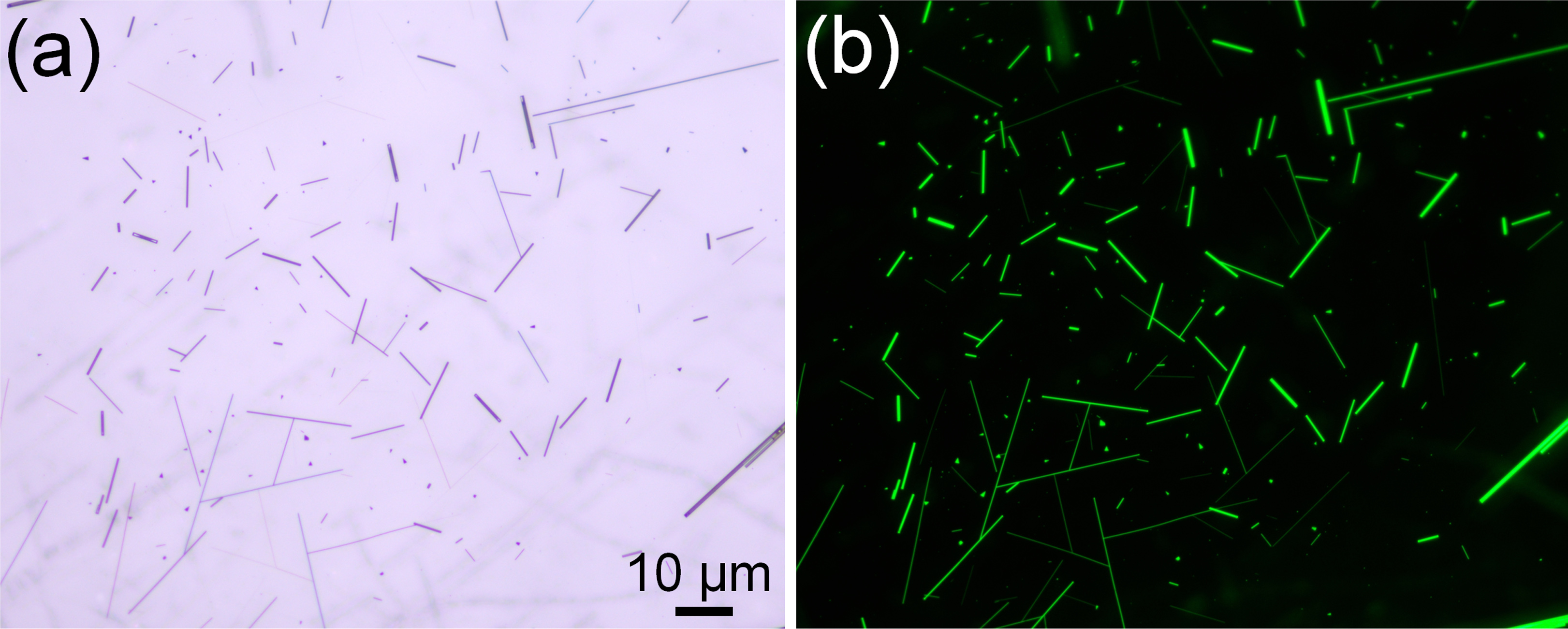


**Figure S1.** (a) Optical photographs and (b) dark-field fluorescence images of CPB microwires fabricated by CVD.

**
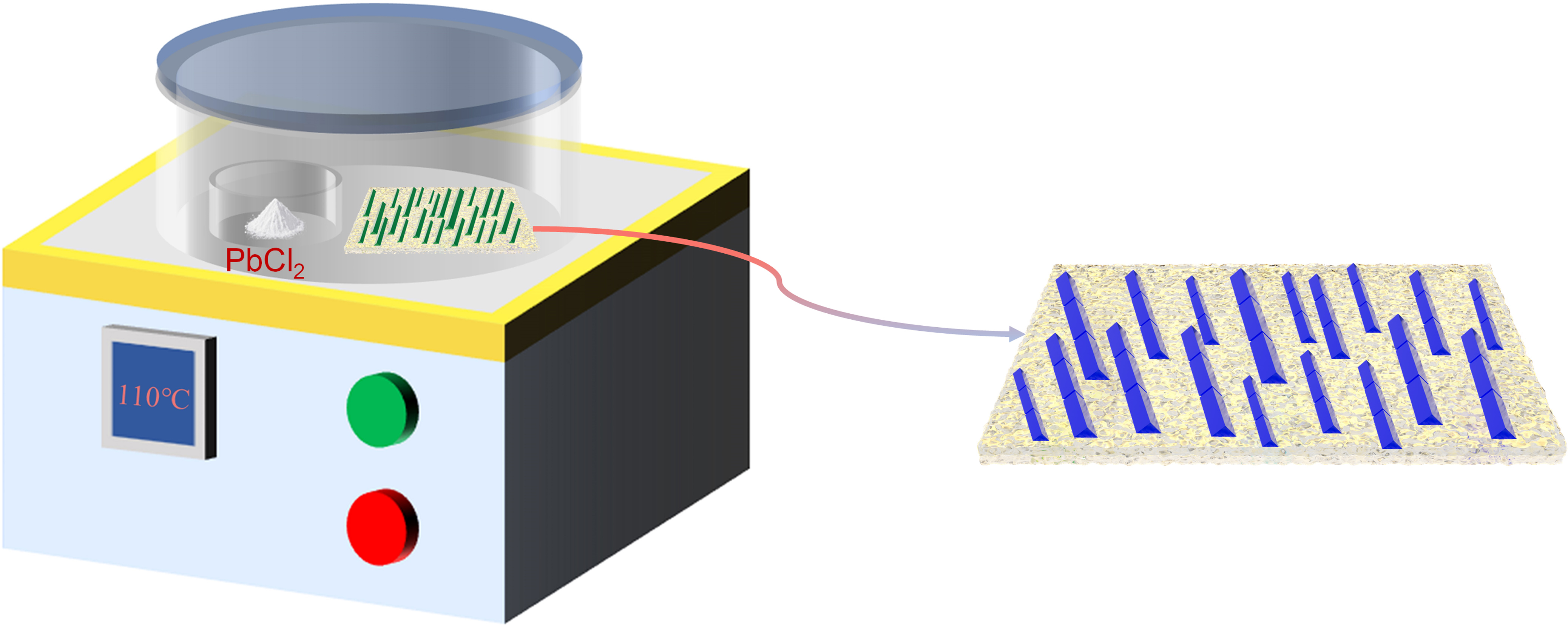
**

**Figure S2.** Schematic diagram of the fabrication setup for CPCB microwires.

**
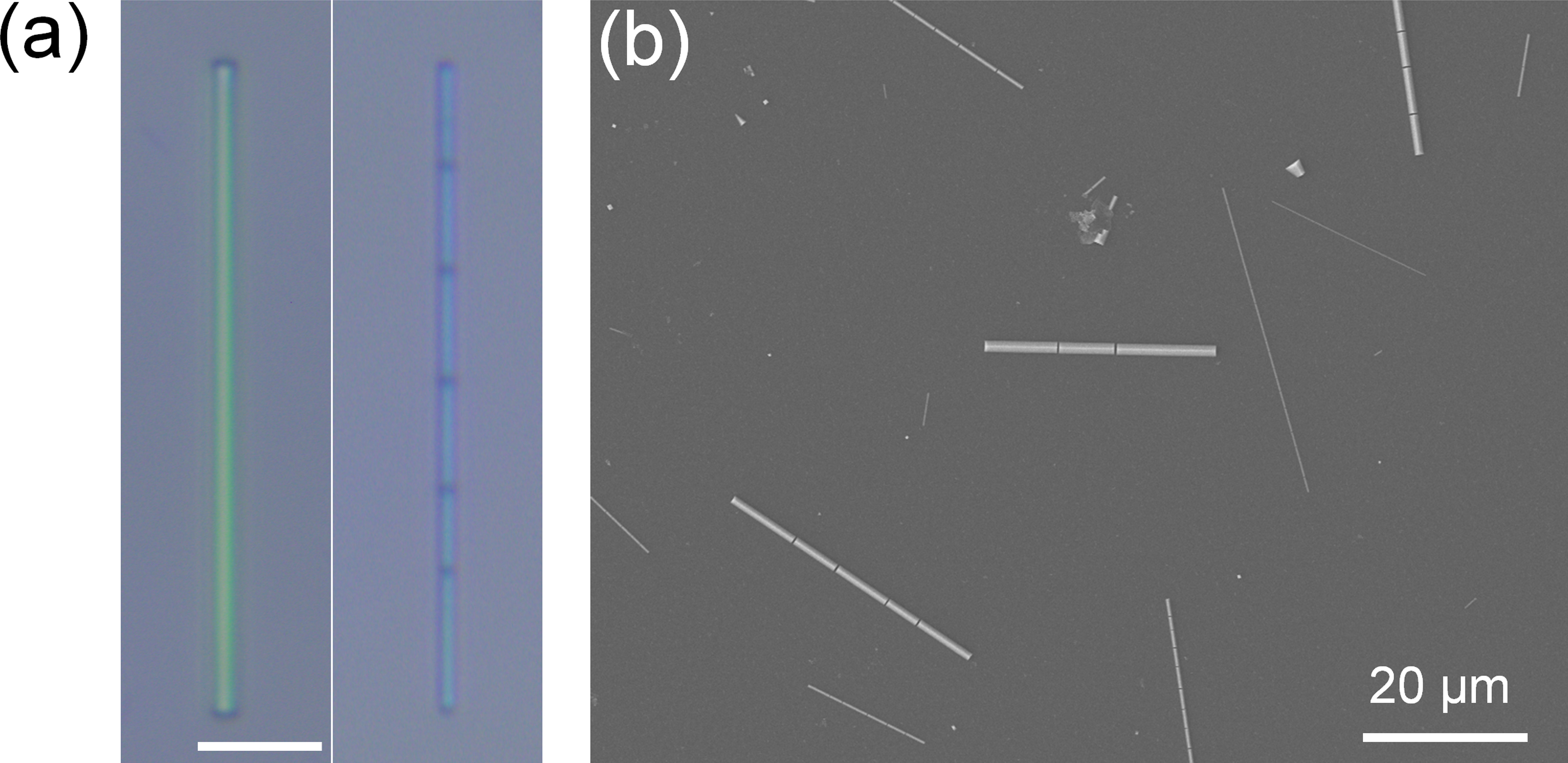
**

**Figure S3.** (a) Optical images of microwires before and after anion exchange, scale bar: 5 μm. (b) SEM image of the microwire after anion exchange.


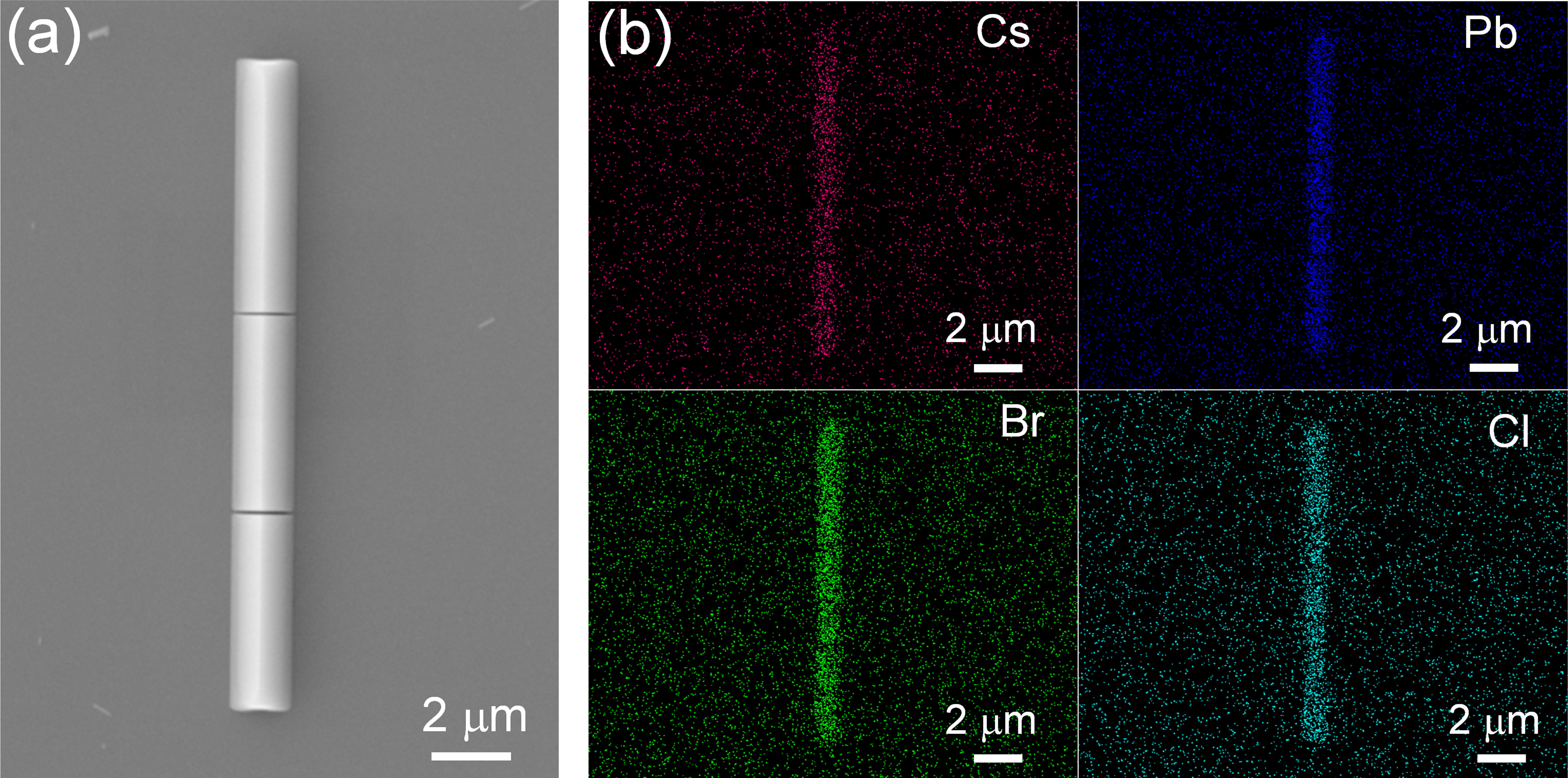


**Figure S4.** (a) SEM image and (b) EDS elemental mapping of CPCB microwires.

**
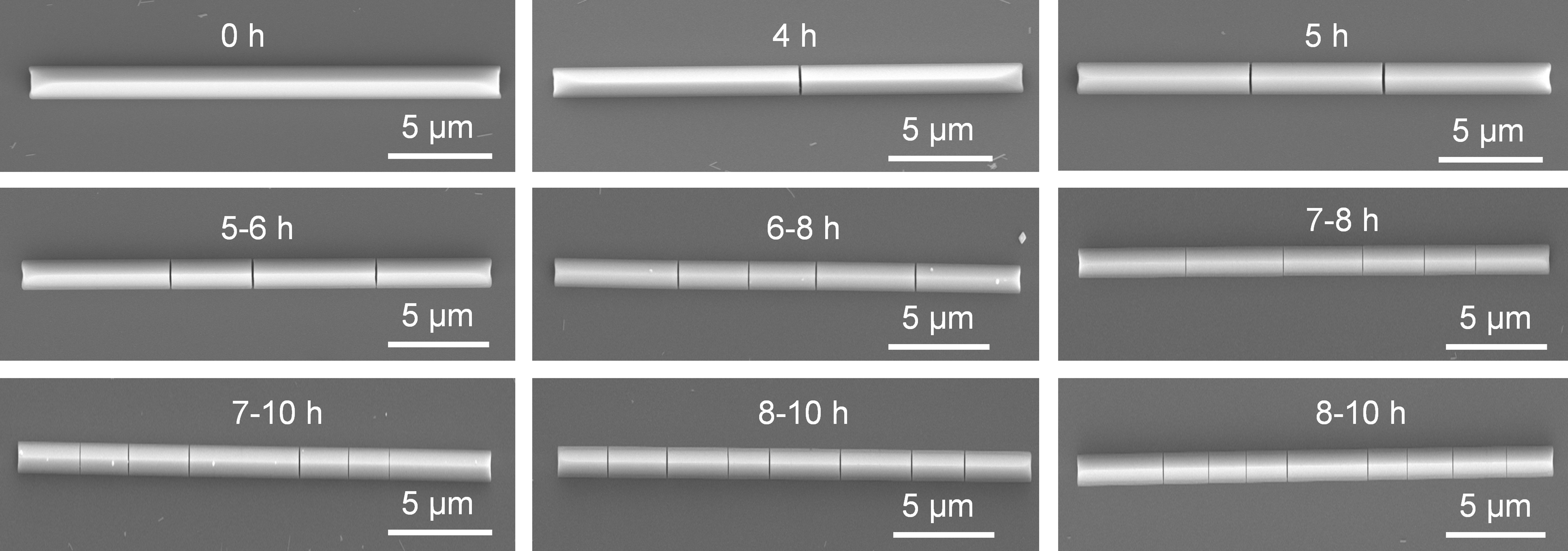
**

**Figure S5.** For microwires with similar dimensions, slits of different numbers can be achieved under different anion exchange times.


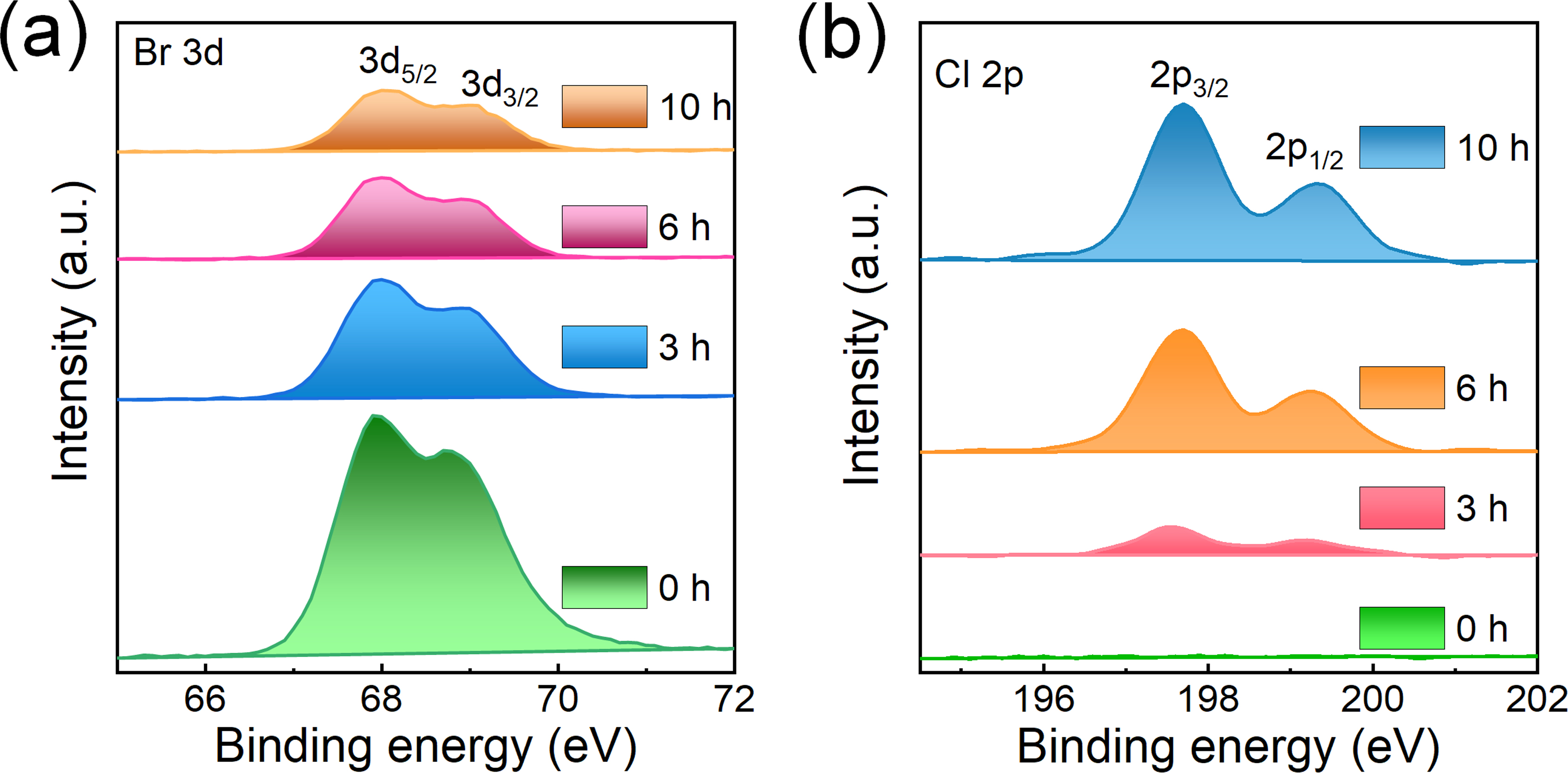


**Figure S6.** (a) Br 3d and (b) Cl 2p XPS spectra of the CPCB microwires.


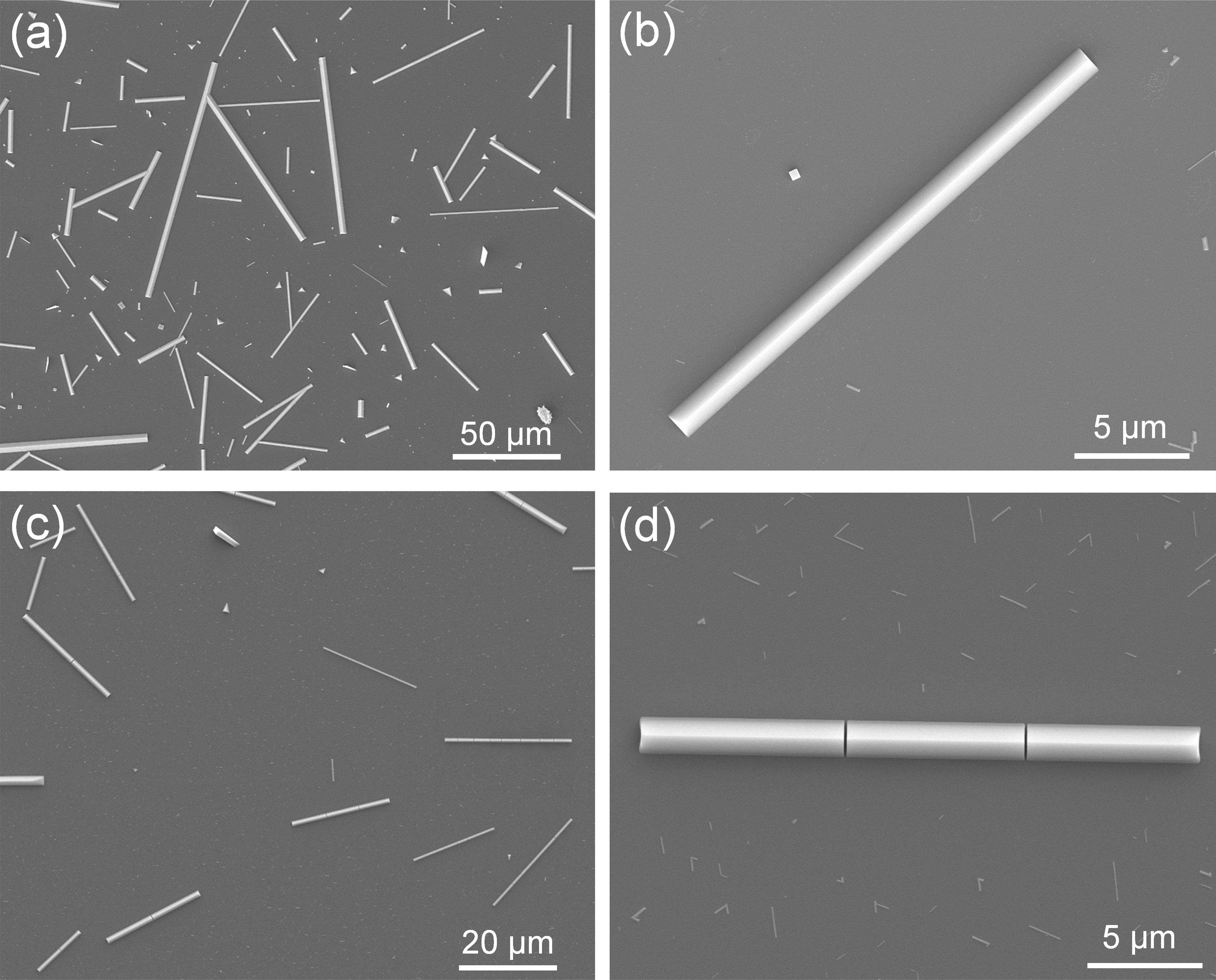


**Figure S7.** SEM images of microwires on the mica substrate (a) without and (c) with anion exchange treatment. SEM images of a single microwire (b) without and (d) with anion exchange treatment.


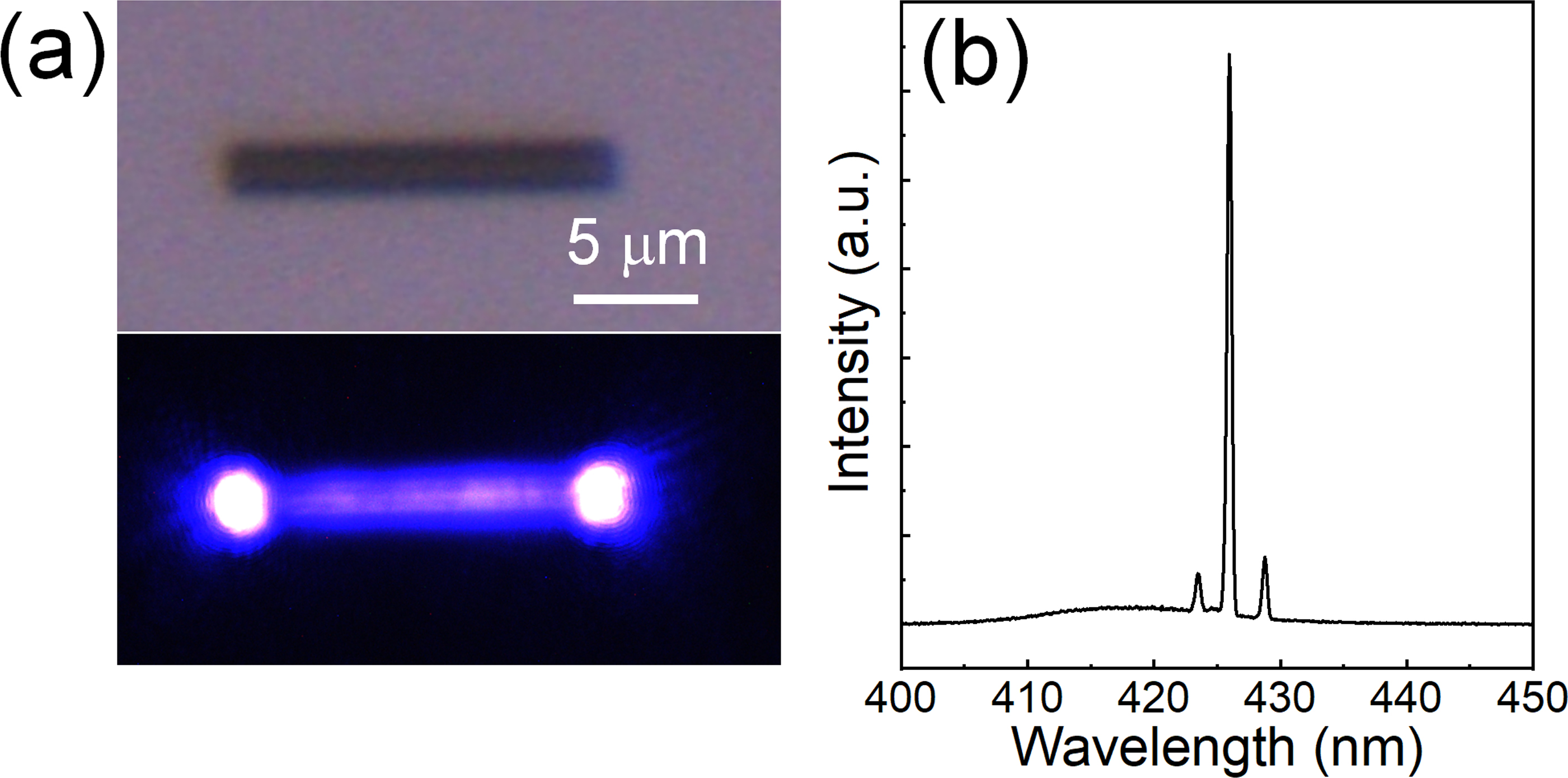


**Figure S8.** (a) Optical photograph and optically pumped dark-field luminescence image of as-prepared CsPbCl_3_ microwires via CVD. (b) Lasing spectrum of CsPbCl_3_ microwires.


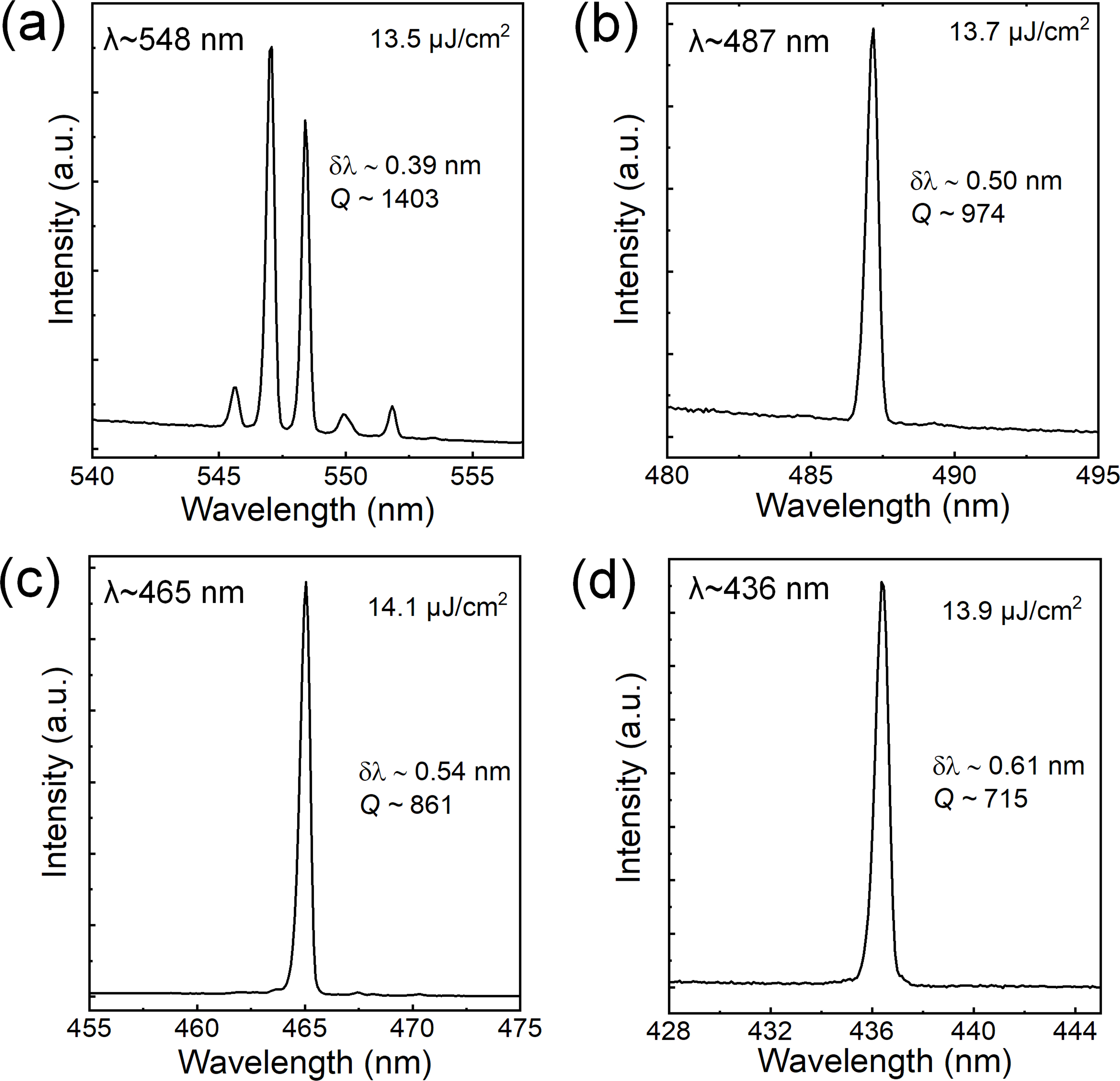


**Figure S9.** Lasing spectra of the sample at a fixed pump energy density with emission wavelengths centered at (a) 548 nm, (b) 487 nm, (c) 465 nm, and (d) 436 nm, respectively.


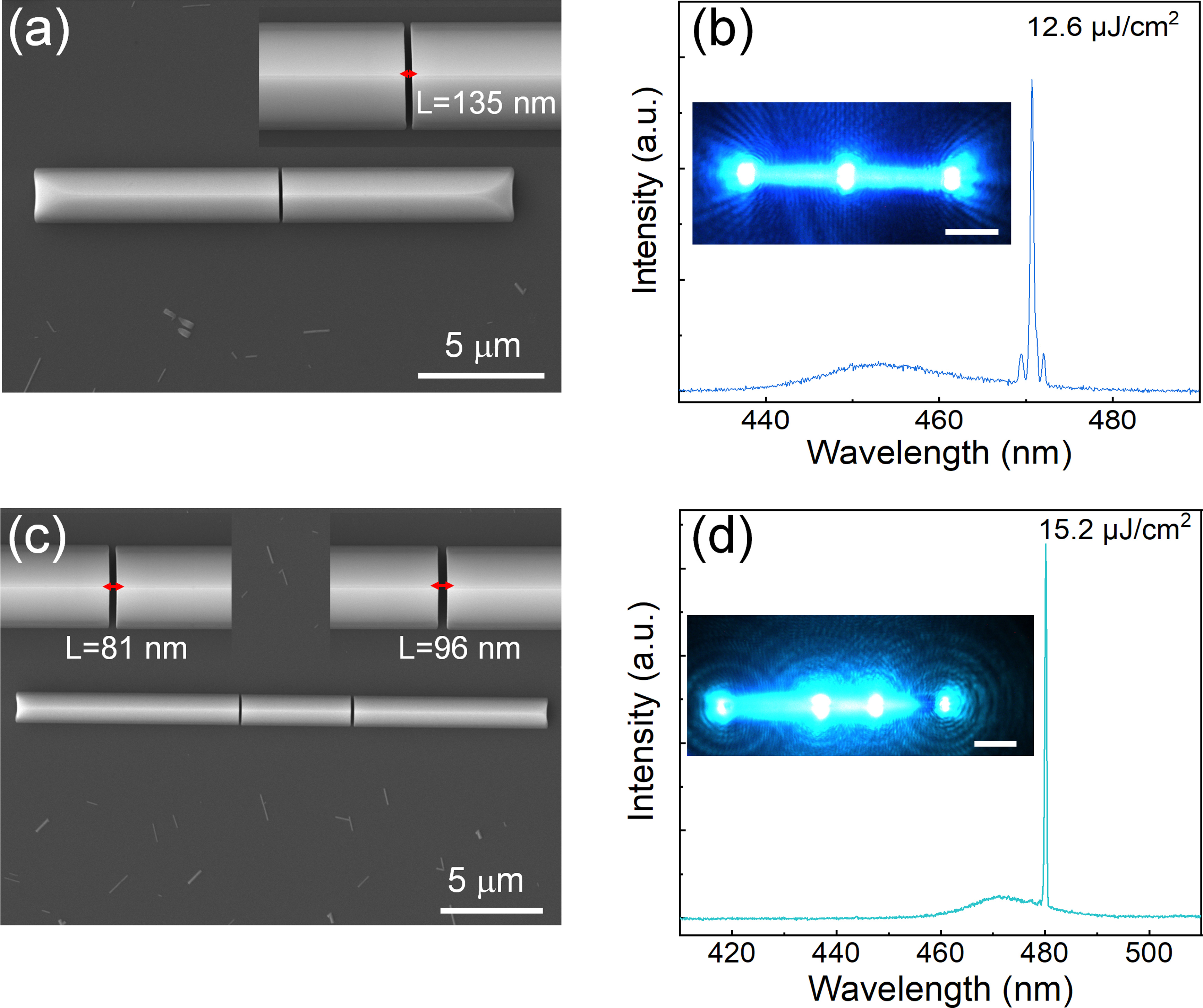


**Figure S10.** (a) SEM image of the microwire with one slit. (b) Dark-field luminescence image and corresponding lasing spectrum of the microwire with one slit, scale bar: 5 μm. (c) SEM image of the microwire with two slits. (d) Dark-field luminescence image and corresponding lasing spectrum of the microwire with two slits, scale bar: 5 μm.


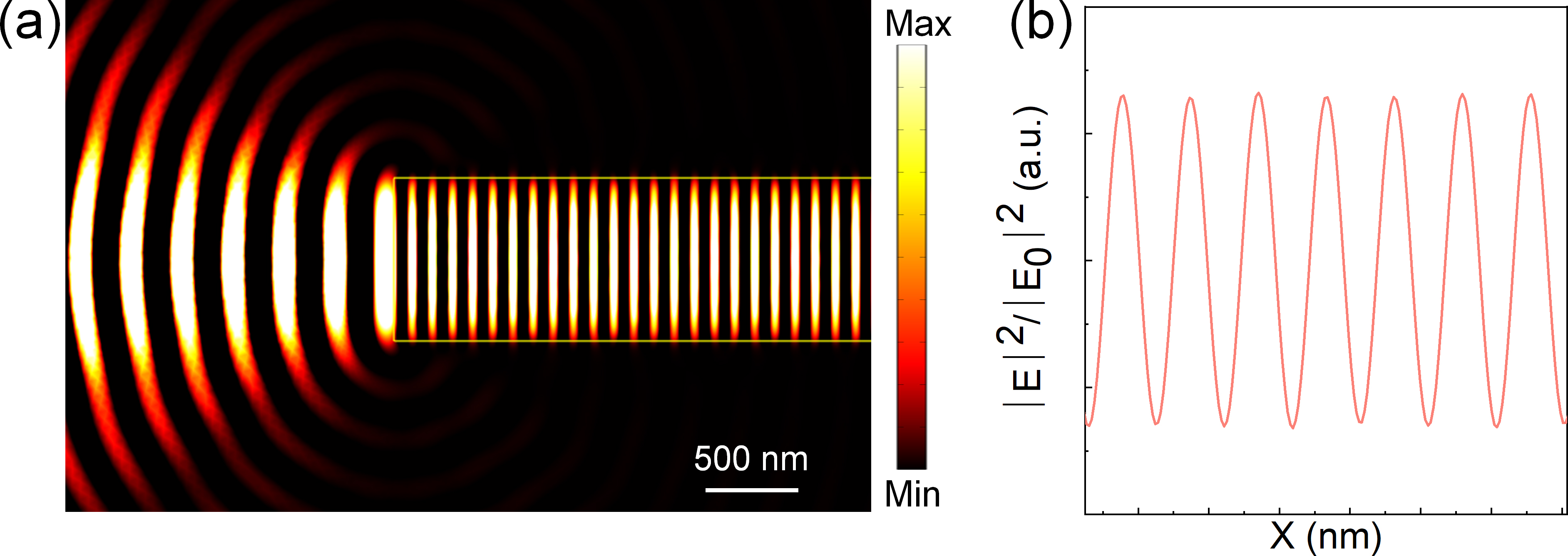


**Figure S11.** (a) Enlarged electric field distribution at the end face of the slit‑free microwire.(b) Enlarged spectral profile of the field distribution.


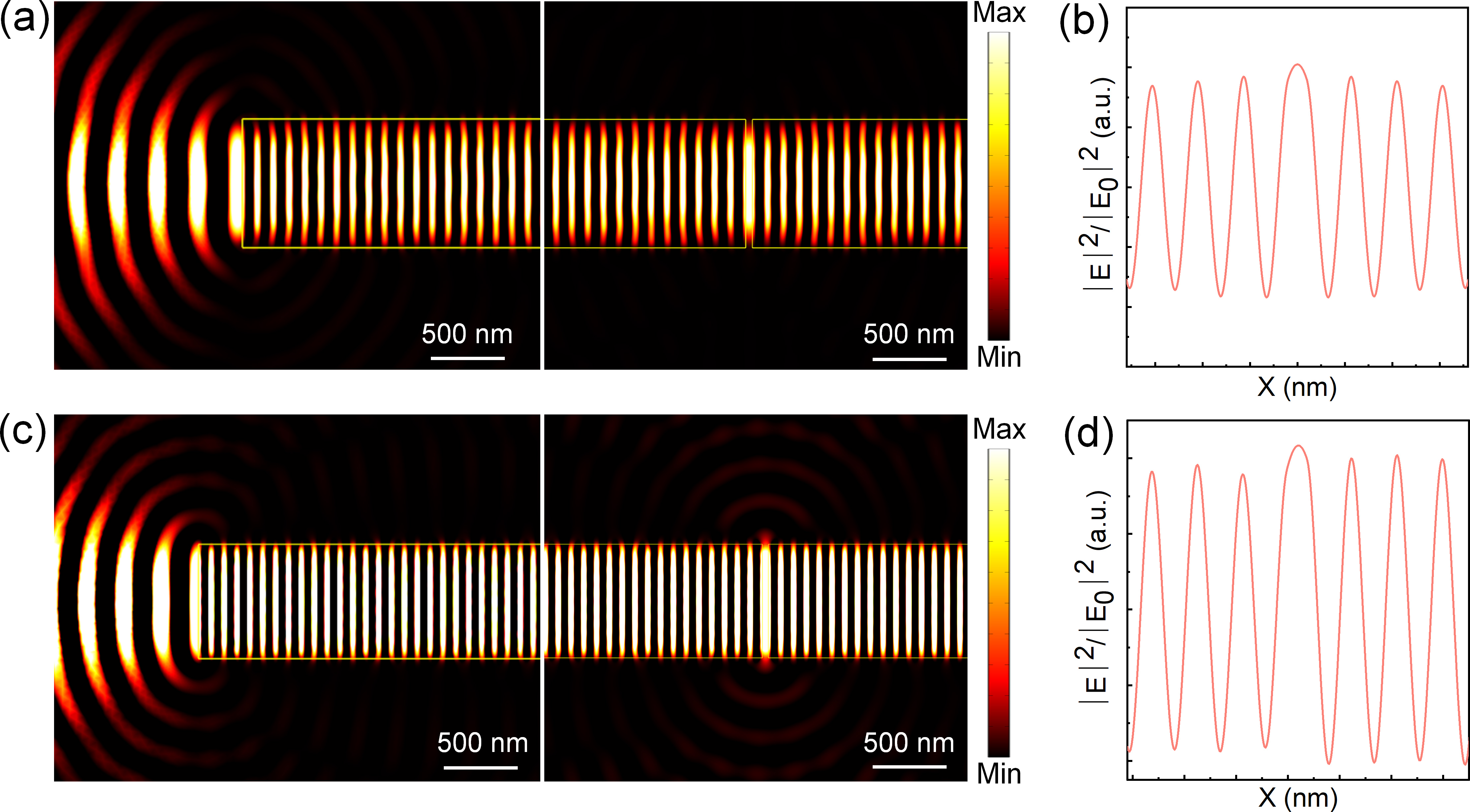


**Figure S12.** (a) Enlarged electric field distribution at the end face of the microwire with one slit, and (b) enlarged spectral profile of the field distribution at the slit. (c) Enlarged electric field distribution at the end face of the microwire with two slits, and (d) enlarged spectral profile of the field distribution at the slits.


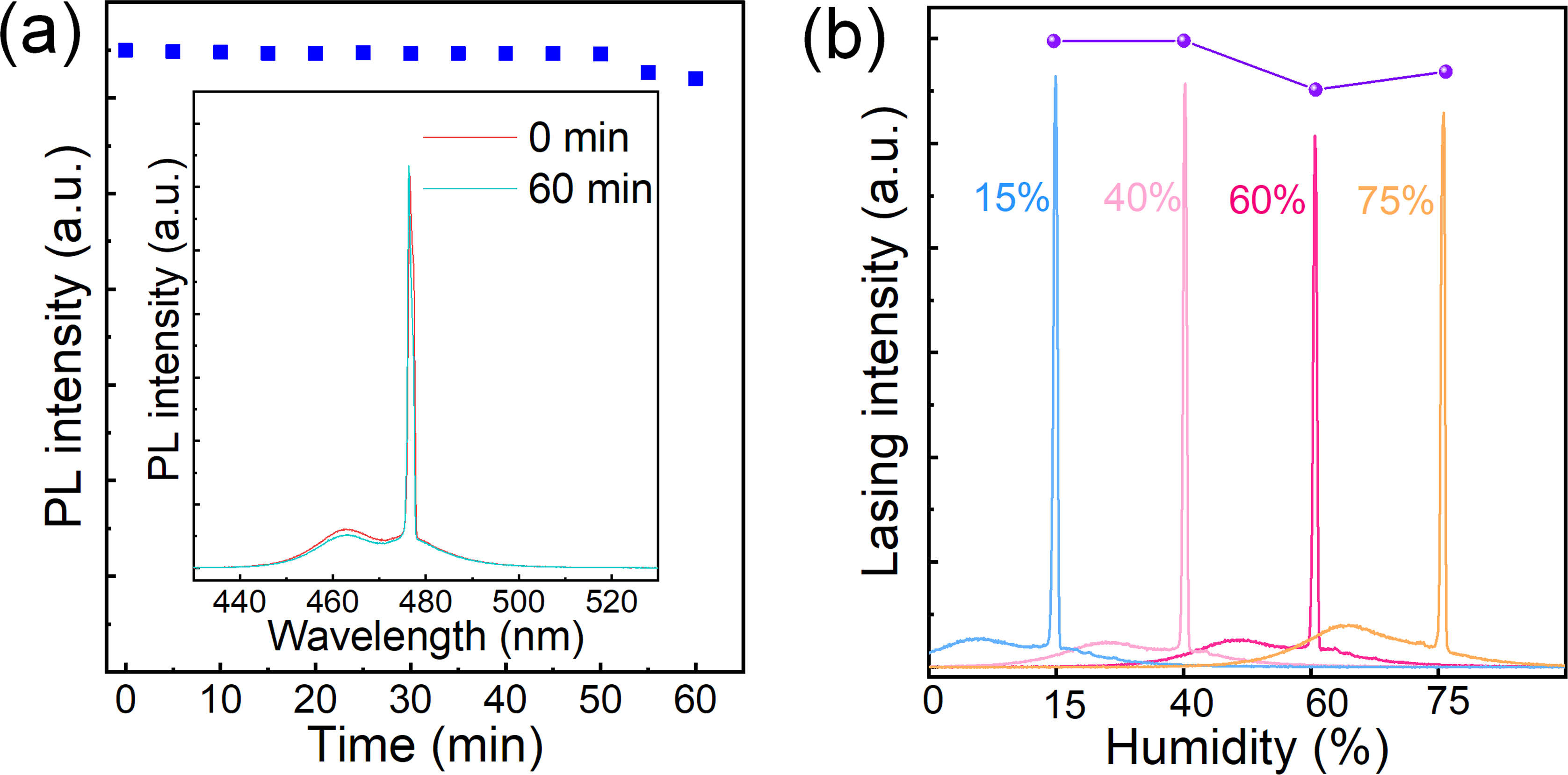


**Figure S13.** (a) Evolution of lasing intensity of the sample with excitation time, the inset shows the lasing spectra collected at 0 min and 60 min. (b) Lasing spectra of the sample after storage for 150 min under different humidity conditions.
